# Composites of aluminum alloy and magnesium alloy with graphite showing low thermal expansion and high specific thermal conductivity

**DOI:** 10.1080/14686996.2017.1286222

**Published:** 2017-03-08

**Authors:** Valerio Oddone, Benji Boerner, Stephanie Reich

**Affiliations:** ^a^ Department of Physics, Freie Universität Berlin, Berlin, Germany

**Keywords:** Metal matrix composites, lightweight, graphite, thermal conductivity, thermal expansion, sintering, 60 New topics / Others, 103 Composites, 104 Carbon and related materials, 201 Electronics / Semiconductor / TCOs, 210 Thermoelectronics / Thermal transport / insulators, 304 Powder processing / Sintering

## Abstract

High thermal conductivity, low thermal expansion and low density are three important features in novel materials for high performance electronics, mobile applications and aerospace. Spark plasma sintering was used to produce light metal–graphite composites with an excellent combination of these three properties. By adding up to 50 vol.% of macroscopic graphite flakes, the thermal expansion coefficient of magnesium and aluminum alloys was tuned down to zero or negative values, while the specific thermal conductivity was over four times higher than in copper. No degradation of the samples was observed after thermal stress tests and thermal cycling. Tensile strength and hardness measurements proved sufficient mechanical stability for most thermal management applications. For the production of the alloys, both prealloyed powders and elemental mixtures were used; the addition of trace elements to cope with the oxidation of the powders was studied.

## Graphical abstract

**Figure:**
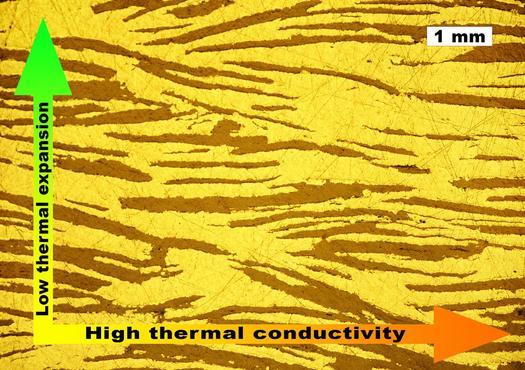

## 1. Introduction

The low thermal expansion of typical semiconducting materials found in electronics induces mechanical stress at the interface with conventional metallic heat sinks [1,2]. Graphene is well known for its extremely high thermal conductivity [3] (TC) and negative coefficient of thermal expansion (CTE) over a broad temperature range [4]; therefore, several studies used it as a filler for thermal-management composite materials [5,6]. While polymer matrices have too low TC for the production of high performance thermal management composites, metal matrices are promising for this type of application, but appropriate production techniques need to be developed [7]. The high TC of metals is due to the high density of free electrons. In graphene and carbon based materials, in contrast, thermal transport is dominated by phonons. This results in a large interface thermal resistance (Kapitza resistance) between filler and metal matrix and strongly decreases the TC of the composite [6]. Crystalline graphite platelets and highly oriented pyrolytic graphite have lower TC than graphene, but perform better as fillers in composites, due to the lower surface-to-volume ratio resulting in fewer interfaces [8–12].

Polymer matrices can easily be solved to a viscous phase, easing the preparation of homogeneous matrix–filler mixtures. This is impossible in the metal melt, where the components separate by density. Powder metallurgy and pressure infiltration of porous fillers [13] were proven to be well-suited methods to produce homogeneously mixed metal matrix composites. The best results were obtained by spark plasma sintering (SPS) [11,14–16]. The short process time minimizes the chemical reactions between metal and filler, reducing for instance the formation of deleterious carbides in metal–carbon composites [11], and avoids the growth of larger crystals in the matrix [15,16], enhancing the mechanical stability. The simultaneous compression and heating of the SPS, similarly to other hot-pressing techniques, minimizes the porosity of the sintered sample [14,17]. Moreover, the current flowing through the sample was shown to have a positive effect on mass transport [14]. It allows the sintering of alloys, starting from pure metals instead of prealloyed powders. These mixtures are cheaper to produce and, for instance in the case of aluminum alloys, are less sensitive to oxidation. Finally, SPS is more suited than conventional sintering techniques if metallic powders with an oxide layer are used [18].

Copper, with a TC as high as 400 W m^–1^ K^–1^, a CTE of 17 ppm K^–1^ and a density of 8900 kg m^–^³, is the most commonly used material in heat sinks. In previous works, copper–graphite composites with improved TC (up to 500 W m^–1^ K^–1^), reduced thermal expansion (as low as 2 ppm K^–1^) and lower density (5500 kg m^–^³) were produced [12]. Here we report new graphite-reinforced composites using light aluminum alloys and magnesium alloys as matrix with density below 2000 kg m^–^³. Though these metals have much lower TC than copper, in the obtained metal–graphite composites values up to 380 W m^–1^ K^–1^ were measured. The CTE was reduced to –10 ppm K^–1^. Most interestingly for mobile and aerospace applications, the low density of these composites led to a specific thermal conductivity over four times lower than in copper.

## 2. Materials and methods

Composites of metals and graphite were produced by SPS. Several metal matrices were sintered with different parameters (temperature, heating rate and time) and graphite concentrations of up to 65% in volume.

### 2.1. Preparations of powder mixtures

The metal matrices were powders of pure aluminum (45 μm, 99%) and pure magnesium (50 μm, 99.9%), prealloyed powders (aluminum alloys: Al7075, i.e. Al-5.8Zn-2.3 Mg-1.4Cu, and Al2024, i.e. Al-4.4Cu-1.4 Mg-0.5Mn, 25 μm; magnesium alloy: AZ31, i.e. Mg-3Al-1Zn) and mixtures of metals (Alumix 231, i.e. Al-2.5Cu-0.5 Mg-14Si or AM231, and Alumix 431, i.e. Al-1.5Cu-2.5 Mg-5.5Zn or AM431, 60 μm; magnesium alloy AZ61, i.e. Mg-6Al-1Zn, mechanically alloyed Mg-0.9Ca). Pure aluminum, aluminum alloys and mixtures were provided by Ecka Granules (Velden, Germany), magnesium by laborladen.de (Hufingen, Germany), AZ31 by MSE-Clausthal (Clausthal, Germany), Mg-0.9Ca was provided by the Helmholtz-Zentrum Geesthacht (Geesthacht, Germany) and for further experiments produced by mechanical alloying of Mg and Mg-9.7Ca (20 min, 200 rpm ball milling with cyclohexane in Ar atmosphere), AZ61 was mixed in our lab from its components. Large graphite flakes (~10 μm × 500 μm) were provided by NGS Naturgraphit (Leinburg, Germany) and Graphene Supermarket (Calverton, NY, USA). The powders were mixed with graphite flakes under an argon atmosphere by hand (preferred) or ball milling. Ball milling was discarded due to the abrasion of the graphite flakes and the self-ignition risk of the as-prepared powder when exposed to air.

### 2.2. Sample preparation

All samples were prepared by SPS in a Dr. SinterLab Jr. 211Lx (Fuji Electronic Industrial Co. Ltd., Osaka, Japan)*.* The sintering temperature was chosen just below the melting point of the metal matrix, which is essential for powders with a strong oxide layer [18,19]. Pure metals were sintered at 600°C, alloys below 5% solutes at 550°C, alloys above 5% at 500°C. We used heating rates of 50 K min^–1^ and 100 K min^–1^, holding times of 2–10 min and pressures of 45 and 50 MPa. The process was performed in a vacuum of 1 Pa. Most samples with alloy matrix received after sintering a T6 thermal treatment for mechanical strengthening (2 h solution heat treatment, quenching in cold water, 18 h artificial aging; for Al7075, Alumix 431 and 231 heat treatment at 450°C, aging at 160°C; for Al2024 470°C and 190°C; for AZ31 400°C and 160°C). Samples of Mg-0.9Ca were annealed for 2 h at 350°C. Few copper–graphite samples were prepared for the Brinell hardness measurements (Section 2.4) according to the parameters described by Firkowska et al. [12]. The uniaxial pressure during SPS orients the graphite flakes in the composites (see Abstract Figure), giving rise to anisotropic properties [17]. We define the direction parallel to the flakes and perpendicular to the pressing axis as *in*-*plane,* the direction parallel to the pressing and perpendicular to the flakes as *through*-*plane.*

### 2.3. Thermal characterization and thermal stress tests

Thermal diffusivity was measured by the flash method with a Netzsch LFA447 NanoFlash (Netzsch, Selb, Germany) for samples with 25 mm diameter and 1–2 mm thickness, whereby the graphite flakes are oriented in the plane. Measurements were performed at room temperature as well as temperature dependent up to 200°C. Through-plane measurements were performed directly. The in-plane measurement was performed by radial heat flow: a special sample holder was used in which the sample is heated in the central part of the front side and the temperature rise is measured close to the edges on the rear side [20,21]. With known through-plane thermal diffusivity, the in-plane thermal diffusivity is evaluated numerically. The TC was determined by multiplying the thermal diffusivity with the density (determined by geometric and Archimedes methods) and the specific heat capacity. Starting from literature values for the specific heat capacities of the matrix *c*_*m*_ and of graphite *c*_*g*_ = 0.702 J kg^–1^ K^–1^, we determined the specific heat capacity of the composite *c*_*c*_ by rule of mixture by mass according to:

$$c_{c}=\;c_{m}\cdot w_{m}+\;c_{g}\cdot w_{g}$$

with the mass concentrations *w*_*m*_ and *w*_*g*_ *.* The thermal diffusivities, the heat capacities (*c*_*m*_) and the measured densities are listed together with the TC results in Table 1. Previous studies have shown that the heat capacity in SPS metal–matrix composites follows the rule of mixture.

**Table 1.** Density (ϱ, kg m^–3^), specific heat capacity (c, J kg^–1^ K^–1^), thermal diffusivity (α, mm^2^ s^–1^) and thermal conductivity (TC, W m^–1^ K^–1^) of metal–graphite composites with different aluminum and magnesium alloys for 50% volume concentration of graphite (Gr), compared with the matrix, copper and copper–graphite composites. Values for thermal diffusivity and conductivity of the composites are given in-plane (IP) and through-plane (TP).

	Cu [12]	Al	Al2024	Al7075	AM431	AM231	Mg	Mg-0.9Ca	AZ31	AZ61
0 vol.% Gr										
ϱ	8370	2650	2750	2800	2770	2640	1670	1720	1740	1880
c	382	897	875	862	864	858	1020	1019	1000	990
α	106	79	54	40	57	60	70	75	40	20
TC	340	187	129	97	137	135	120	132	70	38
50 vol.% Gr										
ϱ	5310	2370	2350	2270	2370	2340	1810	1840	1810	2030
c	446	809	799	792	764	789	845	845	836	858
α IP	211	203	186	184	194	187	222	233	169	162
α TP	30	21	24	21	24	23	23	24	22	25
TC IP	500	390	350	330	351	345	340	362	255	282
TC TP	70	40	45	38	44	42	35	37	33	44

The CTE was determined by dilatometry with a dilatometer L75XH1000 from Linseis (Selb, Germany) Samples with 6 mm diameter and 4–6 mm thickness were measured. Four cycles from 50°C and 150°C with heating/cooling rates of 0.3 K min^–1^ were performed. This temperature range was chosen as a typical operating condition. The mean value between 90°C and 110°C was recorded for the evaluation. A T6 thermal treatment was performed to the alloyed samples before dilatometry. For a thermal stress test 10 cycles between 30°C and 180°C with 1 K min^–1^ were performed with the dilatometer. Additionally, the samples were cooled down to –196°C in liquid nitrogen to verify that the extreme CTE mismatch stress between metal matrix and graphite leaves the inner structure of the sample intact. The integrity of the samples after the thermal stress tests was confirmed by dilatometry and flash measurements.

### 2.4. Mechanical characterization

**Figure 1.**
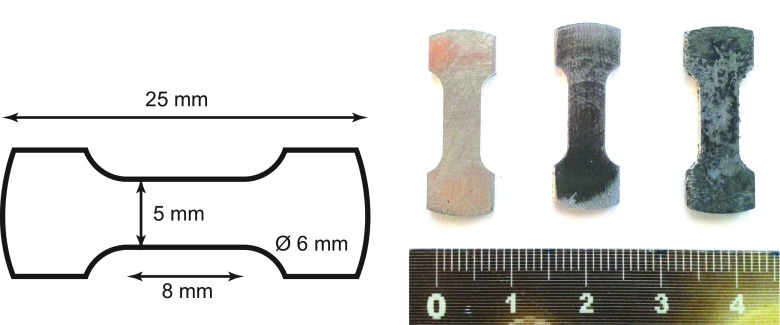
Dog-bone shaped sample for tensile tests.

Tensile strength was measured with a Zwick Z010 (Ulm, Germany) tensile tester*.* The samples used for thermal characterization (25 mm diameter, 1–2 mm thickness) were milled to a dog-bone shape with a gage length of 8 mm and width of 5 mm (Figure 1). Hardness measurements are usually easier than strength measurements, since they have no special requirements on the sample shape. They provide information about the tensile strength as well [22,23]. The Brinell hardness was measured with a 13.5 mm hardened steel sphere as an indenter. Samples with 25 mm diameter and 4–8 mm thickness were indented; a force of 2 kN was applied to the sphere along the through-plane axis. The diameter of the indentation was measured by high-resolution photography.

## 3. Results and discussion

### 3.1. Thermal conductivity

Thermal conductivity was used as the primary parameter for the optimization of the sintering process, since it indirectly gives information about the porosity of the material and the presence of interfaces, e.g. because of the oxide layer on the powders. The experiments clearly showed the size of the graphite flakes to be a fundamental parameter. Large flakes reduce the number of interfaces and the total interfacial thermal resistance, increasing the overall TC. The size of the sample is the limiting factor for the size of the graphite flakes, since large flakes in small samples introduce weak points affecting the mechanical stability.

Magnesium and aluminum in contact with air build a strong oxide layer which inhibits sinter processes [18]. High temperatures and pressures helped in breaking the oxide layer of the metals (higher TC of the sintered samples), while the heating rates and holding times had little influence on the produced samples. This is an important difference to the chemically more stable copper powders, which could be sintered with similar results between 600°C and 900°C. For magnesium powders, the thermal and mechanical properties of the samples could be improved by the addition of 0.9% calcium, as suggested by Wolff et al. [24] and confirmed by the following results. The formation enthalpy of CaO (–635 kJ mol^–1^) is lower than MgO (–602 kJ mol^–1^). The added calcium reduced the oxide layer of the magnesium powder while sintering. Since small amounts of calcium oxide are soluble in the magnesium matrix, they did not deteriorate the properties of the sintered material. For aluminum alloys, the use of pure metal mixtures, such as AM431, instead of the oxidation sensitive prealloyed metals, such as Al7075, led to higher TC and tensile strength. The SPS process formed the alloy despite its short heating time, as demonstrated by the tensile tests in Section 3.3.

**Figure 2.**
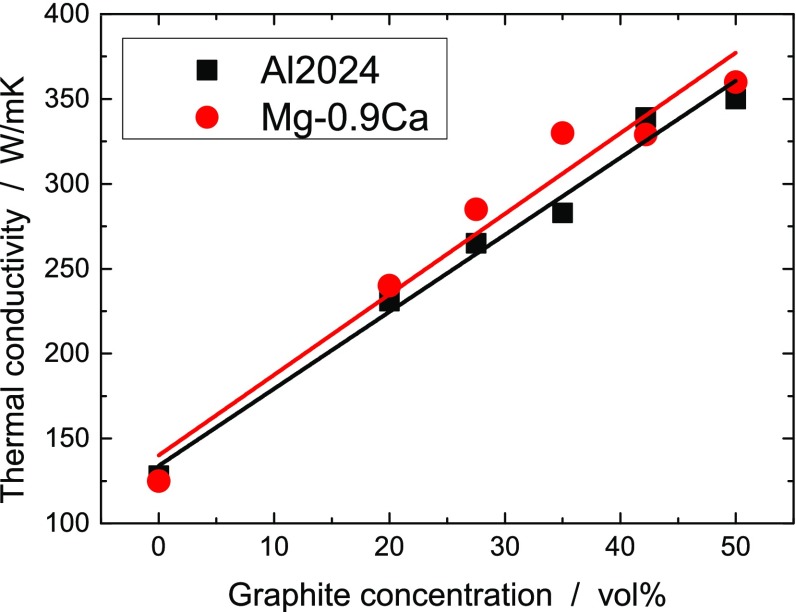
In-plane TC of metal–graphite composites with the aluminum alloy Al2024 and the magnesium alloy Mg-0.9Ca as matrix.

An increase in graphite concentration up to 50 vol.% enhanced the TC in-plane and reduced it through-plane (see Figure 2 and Table 1). The same experiments at 65 vol.% graphite concentration did not follow the trend, probably because of the increasing porosity of the sample, therefore most measurements were performed only up to 50 vol.%. The in-plane TC of graphite–metal composites for the aluminum alloy Al2024 and for the magnesium alloy Mg-0.9Ca as a function of graphite concentration is shown in Figure 2. At 50 vol.% graphite concentration the composites reach the TC of sintered copper. For comparison, cast Al2024 alloy has a TC of 120–190 W m^–1^ K^–1^, depending on the thermal treatment [25].

**Figure 3.**
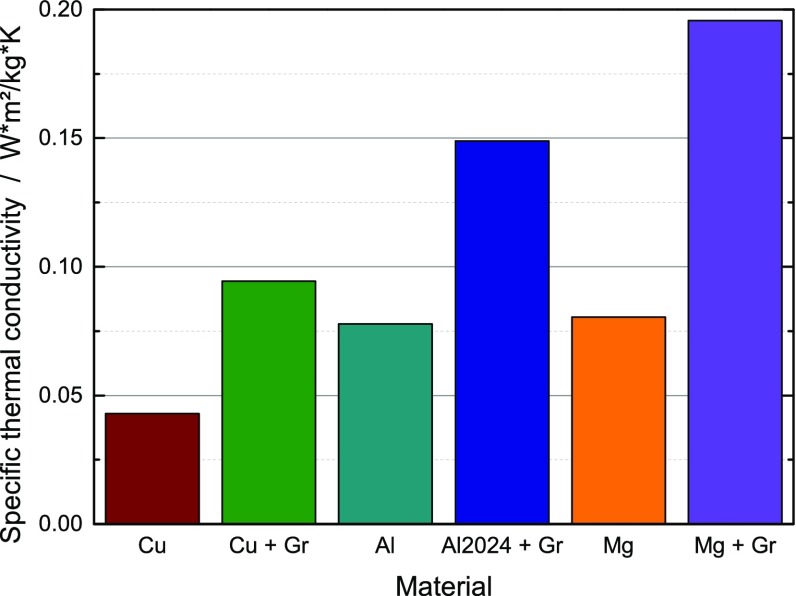
Specific thermal conductivity of copper [12], aluminum and magnesium compared to the sintered metal–graphite composites.

The in-plane thermal conductivities of composites with different metal matrices at 0% and 50% graphite concentration are shown in Table 1 and compared to that of copper. For all matrices we obtained a strong increase of their TC through the addition of graphite fillers. Particularly pronounced is the improvement of the poorly conducting alloys, reaching a maximum increase of 700% when AZ61 was loaded with 50% graphite. We note that all Al and the Mg-0.9Ca composites have a TC close or above that of pure, sintered copper. Oxidation sensitive alloys such as the aluminum alloy Al7075 and the magnesium alloys show lower TC, since the oxide layers inhibit the sinter process. For magnesium, the addition of calcium to reduce the oxide layer enhanced the conductivity by 10–15%. The addition of traces of lead (0.1–1 wt%) to improve the sintering response of Al7075 [26] slightly increased the TC (approximately 5%). The T6 thermal treatment resulted in a 10–20% lower TC probably due to oxidation, therefore the treatment temperature was chosen slightly below the standard practice (up to 490–500°C); thermal shocks in liquid nitrogen did not affect the TC. Similar to pure copper, the TC slightly decreases at higher temperatures. In particular, for Al2024 with 50 vol.% graphite the measured dependency of the TC on temperature between 20°C and 200°C was –0.09% K^–1^. For comparison, for sintered graphite flakes we measured –0.17% K^–1^ and for sintered Al2024 –0.02% K^–1^, so the temperature dependence of the TC in the composite corresponds to the expected value by the rule of mixture, suggesting that the CTE mismatch between matrix and filler does not influence the TC at different temperatures. A prediction of absolute values of TC by composites models can be performed if the thermal conductivity and orientation of the graphite filler are known [12].

Of particular interest for aerospace and mobile applications is the specific thermal conductivity, i.e. TC divided by the density. As shown in Figure 3, the lower TC of samples with aluminum and magnesium matrices compared to copper composites is greatly compensated by the low density of Al and Mg. The specific thermal conductivity of aluminum–graphite composites is more than three times higher than for copper and over four times higher in magnesium–graphite composites.

### 3.2. Coefficient of thermal expansion

The CTE of all samples was reduced by the addition of graphite. Graphite has a CTE of –1 ppm K^–1^ in-plane and 28 ppm K^–1^ through-plane [27]. Remarkably, the CTE of the sintered samples fell down to low or even negative values through-plane, while the in-plane reduction did not exceed 30% in comparison to the metal matrix. Firkowska et al. [12] observed a similar effect in copper–graphite composites with equivalent microstructure and attributed it to an in-plane stretch of the graphite crystal caused by the expansion of the metal matrix. Under consideration of the temperature dependence of the elastic constants and the effects of the cooling after sintering, the model predicts a shrink in the graphite crystal in the through-plane direction. This compensates the overall expansion of the sample along this axis. In our composites the higher CTE of the matrix led to higher in-plane CTE and showed, as expected from the model, a lower through-plane CTE than copper–matrix composites. Many metal matrices used here have too low elasticity moduli to induce strain in the graphite flakes. We suggest a second macroscopic effect playing a role: the graphite flakes get wrinkled by the contracting matrix after sintering and reversibly straightened when heated. In this way, the through-plane thickness of the flakes is reduced leading to the low CTE. An in-depth study of these effects will be subject of future work.

**Figure 4.**
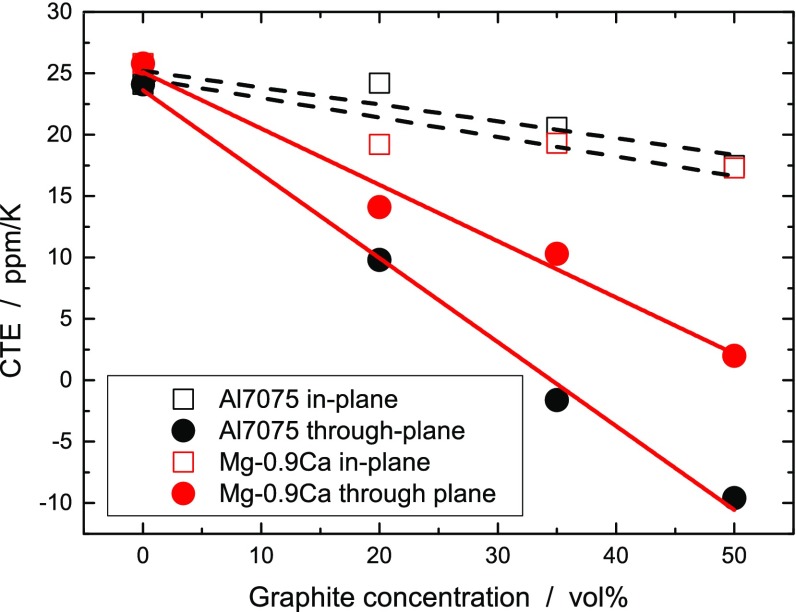
Coefficient of thermal expansion of Al7075-graphite (black) and Mg-0.9Ca-graphite (red) composites as a function of the graphite concentration at 100°C.

The composites are intended for applications with high thermal stress. We therefore report CTE for a temperature of 100°C. The CTE at room temperature is 0–3 ppm K^–1^ lower. Figure 4 shows the change in CTE with increasing graphite concentration for Al7075 and Mg-0.9Ca. The reduction of the through-plane CTE of the metal–graphite composite increased with the Young’s modulus of the matrix (e.g. the Al7075 aluminum alloy). The CTE of other metals and composites at 50 vol.% graphite concentrations is shown in Table 2. Samples for which no T6 treatment was performed showed fluctuating CTE for the first heating/cooling cycles. The samples were observed to be sensitive to mechanical processing. The induced stress may degrade the inner structure to such an extent that the through-plane CTE of the composites increases to the values of the pure metal. This is probably caused by a mechanical damage of the graphite–metal interfaces, reducing the force transfer from the metal matrix to the graphite flakes. Mechanical processing with fast spinning grinding tools minimizes the damage of the inner structure, leading to a minor increase of the CTE (2–6 ppm K^–1^ higher through-plane values). Thermal treatment, thermal cycling and thermal shock in liquid nitrogen did not affect negatively the CTE, proving a good thermal stability of the matrix–graphite coupling.

**Table 2.** CTE (ppm K^–1^) at 100°C of metal–graphite composites with different aluminum and magnesium alloys for 0% and 50% volume concentration of graphite, compared with copper and copper–graphite composites.

Graphite	Cu [12]	Al2024	Al7075	AM431	AM231	Mg-0.9Ca	AZ31
0 vol.%	17	24.7	24.1	24.4	18.5	25.8	25.8
50 vol.% through-plane	2	−7.3	−9.2	−4.2	−2.9	2.1	2.5
50 vol.% in-plane	12	16.6	17.5	20.2	10.9	17.6	17.5

### 3.3. Mechanical properties

**Figure 5.**
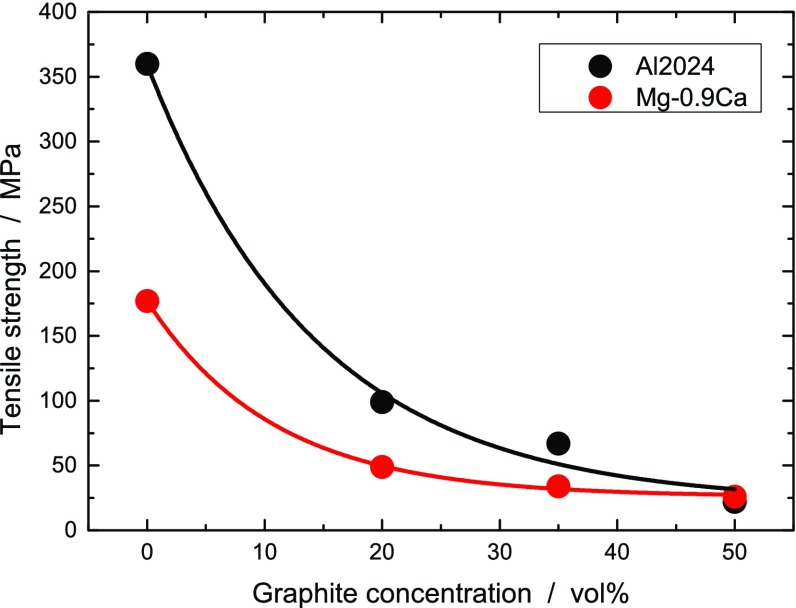
Tensile strength of Al2024-graphite (black) and Mg-0.9Ca-graphite (red) composites as a function of the graphite concentration.

We now examine the mechanical response of the Al and Mg graphite composites. The tensile strength falls exponentially with increasing graphite concentration (see Figure 5). The graphite flakes introduce weak spots in the material. They lead to mechanical failure, in particular, for samples that are of similar dimension than the graphite flakes. The samples used for the tensile tests hardly showed necking, suggesting that the breaking was caused by a few unfavorably aligned graphite flakes.

The tensile strength at 0 vol.% and 50 vol.% for different metal matrices are summarized in Table 3. The samples with 50 vol.% concentration of graphite are sufficiently stable to be used as heat sinks. Since the heat sinks rarely have a structural function, a softer material may even be advantageous for absorbing vibrations and shocks. Samples with graphite concentration up to 65 vol.% could be sintered, but did not show higher TC and no mechanical characterization was performed.

**Table 3.** Tensile strength (MPa) of metal–graphite composites with different aluminum and magnesium alloys for 0% and 50% volume concentration of graphite, compared with copper and copper–graphite composites.

Graphite	Cu	Al	Al2024	Al7075	AM431	AM231	Mg	Mg-0.9Ca	AZ61
0 vol.%	265	142	360	186	422	262	109	177	72
50 vol.%	29	15	22	21	40	42	—	26	11

Samples sintered out of oxidation sensitive powders like the aluminum alloy Al7075 and the magnesium alloy AZ61 have poor properties in comparison with the same materials produced with traditional techniques. For magnesium, the positive effect of the calcium addition as reducing agent of the oxide layer is visible. For aluminum, the alloy Al7075 is clearly outperformed by the mixture AM431, despite their comparable chemical composition. This shows on the one hand the oxidation sensitivity of the Al7075 alloy in comparison with the pure metals in the mixtures, as well as the rapidity of the SPS process in the alloy formation starting from pure metals.

**Figure 6.**
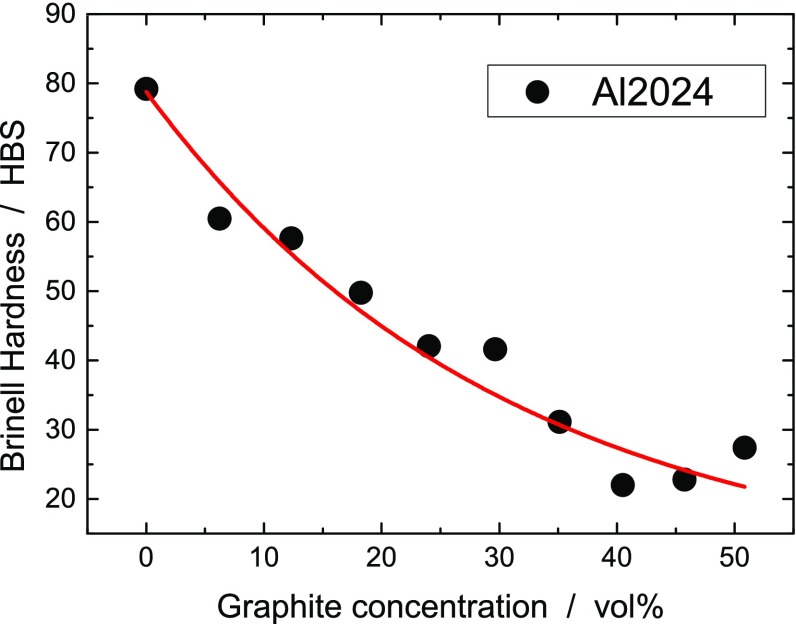
Brinell hardness of Al2024-graphite composites as a function of the graphite concentration.

The Brinell hardness measurements were performed on copper, aluminum, Al7075 and Al2024 composites. Similarly to the tensile strength, hardness exponentially decreased for increasing graphite concentration (see Figure 6). Composites from the Al2024 alloy perform best. The effect of T6 thermal treatment was in this case a constant increase of about 10 points in the HBS scale with a small dependence on the graphite concentration.

## 4. Conclusions

In conclusion, we sintered metal–graphite composites out of aluminum alloys and magnesium alloys by spark plasma sintering. Some composites outperformed copper for specific heat sink applications. The measured thermal conductivity of up to 390 W m^–1^ K^–1^ in our samples is more than two times the value of the metal matrix and approaches the TC of copper. Due to the low density of aluminum, magnesium and graphite, the achieved specific thermal conductivity is four times higher than that of copper. This makes the sintered materials ideal for heat sinks, in particular for mobile or aerospace applications, where a lightweight construction is essential and high thermal fluctuation represent a big stress for electronic components. The reduced mechanical strength of the samples is not a limiting factor as long as the material has no structural function, while the lower elasticity modulus can be exploited for shock absorption. Improved TC and strength were achieved by sintering mixtures of pure elements instead of prealloyed powders.

## Disclosure statement

No potential conflict of interest was reported by the authors.

## Funding

This work was supported by Evonik Stiftung.
